# Capilliposide C from *Lysimachia capillipes* Restores Radiosensitivity in Ionizing Radiation-Resistant Lung Cancer Cells Through Regulation of ERRFI1/EGFR/STAT3 Signaling Pathway

**DOI:** 10.3389/fonc.2021.644117

**Published:** 2021-04-01

**Authors:** Kan Wu, Xueqin Chen, Jianguo Feng, Shirong Zhang, Yasi Xu, Jingjing Zhang, Qiong Wu, Mingliang You, Bing Xia, Shenglin Ma

**Affiliations:** ^1^ Department of Thoracic Oncology, Key Laboratory of Clinical Cancer Pharmacology and Toxicology Research of Zhejiang Province, Affiliated Hangzhou Cancer Hospital, Zhejiang University School of Medicine, Zhejiang University Cancer Center, Hangzhou, China; ^2^ Department of Thoracic Oncology, Affiliated Hangzhou First People’s Hospital, Zhejiang University School of Medicine, Zhejiang University Cancer Center, Hangzhou, China; ^3^ Zhejiang Cancer Research Institute, Cancer Hospital of the University of Chinese Academy of Sciences, Zhejiang Cancer Hospital, Hangzhou, China; ^4^ Center for Translational Medicine, Affiliated Hangzhou First People’s Hospital, Zhejiang University School of Medicine, Zhejiang University Cancer Center, Hangzhou, China; ^5^ Hangzhou Cancer Institute, Affiliated Hangzhou Cancer Hospital, Zhejiang University School of Medicine, Zhejiang University Cancer Center, Hangzhou, China; ^6^ Department of Oncology, Jiande Second People’s Hospital, Hangzhou, China

**Keywords:** *Lysimachia capillipes* capilliposide C, non-small cell lung cancer, radiosensitivity, erbB receptor feedback inhibitor 1, epidermal growth factor receptor

## Abstract

**Aims:**

Radiation therapy is used as the primary treatment for lung cancer. Unfortunately, radiation resistance remains to be the major clinic problem for lung cancer patients. *Lysimachia capillipes* capilliposide C (LC-C), an extract from LC Hemsl, has demonstrated multiple anti-cancer effects in several types of cancer. Here, we investigated the potential therapeutic impacts of LC-C on radiosensitivity in lung cancer cells and their underlying mechanisms.

**Methods:**

Non-small cell lung cancer cell lines were initially irradiated to generate ionizing radiation (IR)-resistant lung cancer cell lines. RNA-seq analysis was used to examine the whole-transcriptome alteration in IR-resistant lung cancer cells treated with or without LC-C, and the differentially expressed genes with most significance were verified by RT-qPCR. Colony formation assays were performed to determine the effect of LC-C and the target gene ErbB receptor feedback inhibitor 1 (ERRFI1) on radiosensitivity of IR-resistant lung cancer cells. In addition, effects of ERRFI1 on cell cycle distribution, DNA damage repair activity were assessed by flow cytometry and *γ*-H2AX immunofluorescence staining respectively. Western blotting was performed to identify the activation of related signaling pathways. Tumor xenograft experiments were conducted to observe the effect of LC-C and ERRFI1 on radiosensitivity of IR-resistant lung cancer cells *in vivo*.

**Results:**

Compared with parental cells, IR-resistant lung cancer cells were more resistant to radiation. LC-C significantly enhanced the effect of radiation in IR-resistant lung cancer cells both *in vitro* and *in vivo* and validated ERRFI1 as a candidate downstream gene by RNA-seq. Forced expression of ERRFI1 alone could significantly increase the radiosensitivity of IR-resistant lung cancer cells, while silencing of ERRFI1 attenuated the radiosensitizing function of LC-C. Accordingly, LC-C and ERRFI1 effectively inhibited IR-induced DNA damage repair, and ERRFI1 significantly induced G2/M checkpoint arrest. Additional investigations revealed that down-regulation of EGFR/STAT3 pathway played an important role in radiosensitization between ERRFI1 and LC-C. Furthermore, the high expression level of ERRFI1 was associated with high overall survival rates in lung cancer patients.

**Conclusions:**

Treatment of LC-C may serve as a promising therapeutic strategy to overcome the radiation resistance and ERRFI1 may be a potential therapeutic target in NSCLC.

## Introduction

Lung cancer is the leading cause of cancer related death worldwide (1, 2), and non-small cell lung cancer (NSCLC) accounts for approximately 85% of all cases. Radiation therapy serves as an important modality for the treatment of NSCLC. Despite major advances in the field of radiotherapy technology during the past decades (3, 4), the prognosis for 5-year survival rate in patients with inoperable locally advanced NSCLC is still poor (approximately 12–24%) (5) due to radioresistance related local recurrence (6). Increasing radiation dose is an important strategy for overcoming radiation tolerance; however, the risk of normal tissue toxicity always limits the dose escalation (7, 8). The mechanism of radioresistance has not been well clarified yet, and effective radiosensitization strategies are urgently needed in the clinic. Recent radiosensitization research has focused on compounds that improve tumor oxygenation (9), interfere with microtubules (10), and activate caspases (11), but the toxicity of most radiosensitizers hampers their clinical application (12). It is therefore crucial to find new therapeutic targets and/or drugs to overcome radioresistance without increasing the adverse effects, and natural products have been considered as promising candidates of radiosensitizers (13, 14).

*Lysimachia capillipes* (LC) Hemsl, a traditional medicinal plant that grows in southeastern China, has been used for treating cough, menstrual, rheumatalgia disorder, and carcinomas. In our previous studies, capilliposide extracted from LC has demonstrated therapeutic effects on lung cancer cells (15, 16). Recently, the main bioactive component capilliposide C (LC-C) (**Figure S1**) (17), a pentacyclic triperpenoid compound extracted from LC, has been tested for its anti-cancer effects. The results showed that LC-C can effectively induce cytotoxic cell death of cancer cells *in vitro* without obvious *in vivo* toxic side effects (18, 19). LC-C could induce apoptosis through activation of caspases, down-regulation of Bcl-2, JNK, and P38a/b, and up-regulation of Bax, p-JNK, and p-P38 in prostate cancer cells (19). Furthermore, a study revealed the pharmacologic advantage of LC-C in oxaliplatin-induced cytotoxicity *via* PI3K/AKT/mTOR pathway (19). Apoptosis is an important mechanism of irradiation-induced toxicity, which suggests that LC-C may have the potential to modulate the radiosensitivity. To data, whether LC-C has radiosensitization effect is still unknown. The purpose of this study is to evaluate the therapeutic potential of LC-C as a radiation sensitizer in ionizing radiation-resistant lung cancer cells.

## Materials And Methods

### Cell Lines and Reagents

A549 and H1299 cell lines were purchased from American Type Culture Collection (ATCC, Manassas, VA, USA). RPMI 1640 medium (Gibco, Waltham, MA, USA) plus 10% heat-inactivated fetal bovine serum (FBS, Gibco, Waltham, MA, USA) was used for cell culture. LC-C was gifted by Professor Tian of the Department of Chinese Medicine Sciences & Engineering at Zhejiang University (Hangzhou, Zhejiang, China). The purity of LC-C was measured >98% using the HPLC-ELSD method. The stock concentration of LC-C was 100 mM, and the working solution of LC-C was prepared with RPMI 1640 medium before the experiment.

### Establishment of Ionizing Radiation-Resistant Cell Lines

A549 and H1299 cell lines were used to establish ionizing radiation-resistant lung cancer cell lines (A549-IR/H1299-IR) as described (20). In brief, the A549 and H1299 cells in exponential growth phase were treated with a repeated IR dose of 2 Gy each (RadSource’s RS 2000 Biological Research Irradiator, Shangai Medicilon, Shangai, China) at room temperature and then returned to the incubator. When they reached approximately 90% confluence, the cells were trypsinized and cultured into new dishes. The fractionated irradiations were continued until the total dose reached 80 Gy. The parental cells were trypsinized and passaged under the same conditions without irradiation. The development of radioresistance was evaluated through MTT and colony formation assays.

### MTT Assay

MTT assay was conducted for cell proliferation analysis, as per the manufacturer’s instructions (Sigma, St. Louis, MO, USA). Cells were cultured in 96-well plates at 8 × 10^3^ cells per well and allowed to adhere overnight. Cell viability was tested 24 h after treatment with different concentrations of LC-C. After treatment, cells were incubated with 50 ml of 1 mg/ml MTT in PBS for 3 h. The purple formazan was then solubilized by DMSO and absorbance at 570 nm was determined with a Plus 384 microplate reader (Molecular Devices LLC, Sunnyvale, CA, USA). The IC_30_ value, which was defined as the concentrations of drug with 30% inhibitory potency, was calculated by GraphPad Prism 8.0 software (San Diego, CA, USA).

### Colony Formation Assay

Colony formation assay (21, 22) was conducted in six-well plates. Briefly, cells were exposed to LC-C (IC_30_ value) for 24 h as pretreatment, and then irradiated with different doses (200 MU/min) or left untreated as control. After treatment, 500–8,000 cells were re-plated with fresh medium and left untouched for 7–14 days until the colony formed. Cells were fixed and stained, and colony numbers were counted under the microscope (Sigma, St. Louis, MO, USA).

### RNA-seq Analysis

Total RNA was extracted from A549-IR cells treated with or without LC-C using the TRIzol reagent (Invitrogen, Carlsbad, CA, USA). RNA degradation and contamination were monitored using GeneGreen-stained 1% agarose gels (Invitrogen, Carlsbad, CA, USA), and RNA purity was determined using a NanoPhotometer^®^ spectrophotometer (Implen, Westlake Village, CA, USA). RNA concentration and integrity were measured using the RNA Nano 6000 Assay Kit of a Bioanalyzer 2100 system (Agilent Technologies, Santa Clara, CA, USA). Ribosomal RNA (rRNA) was then removed from total RNA using the RiboMinus Eukaryote Kit (Invitrogen, Carlsbad, CA, USA) and oligonucleotide probes for rRNAs. Sequencing libraries were constructed with the RNA samples by the NEBNext mRNA Library Prep kit (New England BioLabs, Ipswich, MA, UK) and used for sequencing.

### Differential Gene Patterns and Pathway Analysis

The genes with p-value less than 0.05 and fold change greater than 2 were selected as differentially expressed genes across sample groups. KEGG analysis was used to find significantly enriched pathways (23).

### Quantitative Real-Time-PCR

RNA extraction and reverse-transcribed were conducted in A549-IR cell lines treated with or without LC-C with TRIzol reagent (Invitrogen, Carlsbad, CA, USA) and M-MLV Reverse Transcriptase (Promega, Fitchburg, WI, USA), respectively. Quantitative RT-PCR analysis was performed with a SYBR-Green PCR Master Mix Kit (Applied Bioscience, Foster City, CA, USA). *β*-actin was used as control. The PCR primers were designed with Primer Premier Version 5.0 (Premier Biosoft International, Palo Alto, CA, USA), and all sequences of primers were listed in **Table 1**.

**Table 1:** Primer sequences used in the study.

Gene name	Forward primer (5′–3′)	Reverse primer (5′–3′)
*ERRFI1*	*ACCCCTCCACTGACACCCAT*	*CTTCGCCTGCCAGGAACATC*
*GA45A*	*TGCTGCGAGAACGACATCAAC*	*TTTCCCGGCAAAAACAAATAAG*
*PLK3*	*GCCCCCAGCGGAACAGAACC*	*GCACCCACGGAGAAGGAGAAGT*
*BCL10*	*ACAGAAGATTACAGATGAAGTGC*	*TTAGTAGAAAAAAAGGGCGT*
*β-actin*	*AGCACAGAGCCTCGCCTTTGC*	*CTGTAGCCGCGCTCGGTGAG*

ERRFI1, ErbB receptor feedback inhibitor 1; GA45A, growth arrest and DNA damage gene 45A; PLK3, Polo-like kinases 3; BCL10, B cell lymphoma/leukemia-10.

### ERRFI1 Knockdown and Overexpression in A549-IR Cells

To overexpress ERRFI1 in A549-IR cells, an expression construct was generated by subcloning the Coding Sequence (CDS) of human ERRFI1 into a pcDNA3.1 vector (Invitrogen, Carlsbad, CA, USA), and the integrity of the respective plasmid constructs was confirmed by DNA sequencing. ERRFI1 siRNA was designed and purchased from Santa Cruz Biotechnology (Santa Cruz, CA, USA). ERRFI1 overexpressing plasmid, siRNA, and their respective controls were transduced into cells using Lipofectamine 2000 (Invitrogen, Carlsbad, CA, USA). Western blotting was used to validate the changes of protein expression.

### Flow Cytometry Analysis of Cell Cycle

Cell cycle distribution was determined by flow cytometry analysis. Briefly, cells were fixed with 70% ethanol after treatment, and then stained with 50 μg/ml PI (BD Biosciences, San Jose, CA, USA) in the dark for 30 min. Stained cells were analyzed by flow cytometry (FACS Calibur, BD Biosciences, San Jose, CA, USA), and the percentages of cells at G1, S, and G2/M were calculated by the FlowJo software program (TreeStar Inc., Ashland, OR, USA) based on the DNA content.

### Immunofluorescence

Cells grown on glass slide were irradiated and were then washed twice with Ca++/Mg++-free PBS and fixed in 4% paraformaldehyde. Immunofluorescence staining was performed with *γ*-H2AX antibody (rabbit monoclonal, Cell Signaling Technology, Trask Lane Danvers, MA, USA). DAPI (Invitrogen, Carlsbad, CA, USA) was used for nuclear counterstaining. Images were acquired with LSM 510 confocal microscope (Zeiss) and processed by Photoshop (Adobe). At least 100 cells from each experiment were selected at random and were counted to calculate the percentage of cells as “positive” for *γ*-H2A.X foci if they displayed >5 discrete dots in the nuclei.

### Western Blotting

Cells were lysed in RIPA buffer containing HALT inhibitor (Sigma, St. Louis, MO, USA) with mild sonication for indicated western blot assays. The antibodies were from: Abcam (ERRFI1 and EGFR), Cell Signaling Technology (p21, phosphorylation-CDC2 (Try15), phosphorylation-CHK2 (Thr68), phosphorylation-ATM (Ser1981), EGFR, phosphorylation-EGFR (Tyr1068), STAT3, phosphorylation-STAT3 (Tyr705)), and Santa Cruz Biotech (phosphorylation-EGFR). Anti-*β*-actin (Abcam) was included for equivalent protein loading.

### Animal Experiments

All animal work was conducted in accordance with the ethical guidelines of the Animal Care and Use Committee, Zhejiang Chinese Medical University (No. SYXK (Zhe) 2018-0012). Immunocompromised nude mice were purchased from The Shanghai Laboratory Animal Center of Chinese Academy Sciences (Shanghai, China). 5 × 10^6^ A549-IR cells in 0.1 ml 1× HBSS with 50% Matrigel (Corning, USA) were inoculated subcutaneously into the right thigh of 6–8 weeks old female nude mice. When average tumor volume reached an appropriate size range (approximately day 10 after inoculation), mice were randomly grouped into groups of: drug alone (LC-C 25 mg·kg^−1^, every other day, begin on day 1), radiation alone (6 Gy X-rays, given after drug treatment on day 5), combination of LC-C and radiation (LC-C 25 mg·kg^−1^ plus 6 Gy), or administration with physiological saline as control (n = 6 per group). LC-C and physiological saline were intragastric administrated. Tumor growth was tracked with caliper measuring every two days, and tumor volume was calculated according to the formula: length × width^2^ × 0.5. Body weight of these animals was also measured every two days. Total experiment lasted 13 days, and the study would be terminated early if tumor burden exceeds 2 cm or further complications affect animal welfare. Mice were humanely euthanized after experiment terminated, and tumors were excised for further tests.

Xenograft tumors were collected and paraffin-embedded for immunohistochemistry analysis with ERRFI1 antibody (rabbit monoclonal, Abcam, Cambridge, UK).

### Statistical Analysis

GraphPad Prism 8.0 software was used for statistical analyses. Data are presented as the means ± standard deviation. The t-test was used for statistical analysis, and P-value <0.05 was considered statistically significant.

## Results

### LC-C Significantly Enhances the Radiosensitivity of Ionizing Radiation-Resistant Lung Cancer Cells

**Figure 1:**
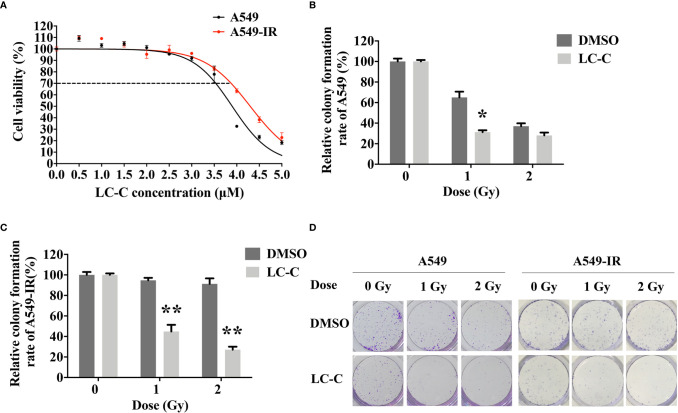
LC-C significantly enhances the radiosensitivity of A549-IR cells. **(A)** MTT assay showing the cell viability of A549-IR cells was slightly higher than A549 cells after treatment with LC-C, and the IC_30_ dose of LC-C in both cells was about 3.5 μM to 4 μM. **(B)** A549 cells were pretreated with LC-C (3.5 μM) or DMSO as control for 24 h and were then exposed to indicated dose of radiation. Colony formation assay showed that A549 cells were sensitive to radiation, and LC-C (3.5 μM) only radiosensitized A549 cells after exposure to low dose radiation (1 Gy). **(C)** A549-IR cells were pretreated with LC-C (3.5 μM) or DMSO as control for 24 h and were then exposed to the indicated dose of radiation. Colony formation assay showed that LC-C significantly radiosensitized A549-IR cells as SF2 reduced from 0.91 ± 0.05 to 0.27± 0.03. **(D)** Representative images showing the colony formation of A549 and A549-IR cells. Results shown are the means ± standard deviation. *Significant difference at P < 0.05 level. **Significant difference at P < 0.01 level.

**Figure 2:**
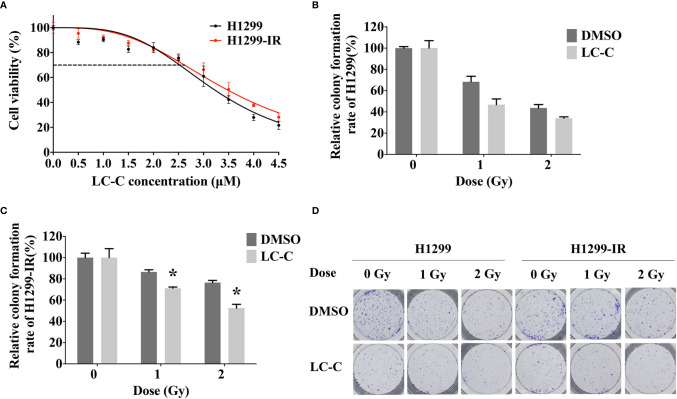
LC-C significantly enhances the radiosensitivity of H1299-IR cells. **(A)** MTT assay showing the IC_30_ dose of LC-C in H1299 and H1299-IR cells was about 2.5 μM to 3 μM. **(B)** H1299 cells were pretreated with LC-C (2.5 μM) or DMSO as control for 24 h and were then exposed to the indicated dose of radiation. Colony formation assay showed that H1299 cells were sensitive to radiation, and there was no obvious radio-sensitization effect of LC-C on H1299 cells. **(C)** H1299-IR cells were pretreated with LC-C (2.5 μM) or DMSO as control for 24 h and were then exposed to the indicated dose of radiation. Colony formation assay showed that LC-C significantly radiosensitized H1299-IR cells as SF2 reduced from 0.77 ± 0.02 to 0.53± 0.04. **(D)** Representative images showing the colony formation of H1299 and H1299-IR cells. Results shown are the means ± standard deviation. *Significant difference at P < 0.05 level.

We first determined the inhibitory effects of LC-C on the cell growth of A549 and A549-IR cells with MTT assay. Our results showed that the cell viability of A549-IR cells was slightly higher than A549 cells after treatment with LC-C, and the IC_30_ dose of LC-C in both cells was about 3.5 μM to 4 μM (**Figure 1A**). To test whether LC-C has the potential to sensitize lung cancer cells to radiation treatment, we treated cells with 3.5 μM LC-C for 24 h and then exposed the cells to different doses of radiation treatment. Colony formation assay showed that A549-IR cells were more resistant to radiation than A549 cells, and LC-C significantly radiosensitized A549-IR cells as the surviving fractions at 2 Gy (SF2) was reduced in the LC-C group compared to the control group (0.27 ± 0.03 *vs*. 0.91 ± 0.05, P < 0.01) (**Figures 1B–D**), indicating a potential radiosensitization effect of LC-C on ionizing radiation-resistant A549-IR cells. In addition, MTT assay showed that the IC_30_ dose of LC-C in H1299 and H1299-IR cells was about 2.5 μM to 3 μM (**Figure 2A**). Furthermore, colony formation assay showed that 2.5 μM LC-C also significantly radiosensitized H1299-IR cells as the SF2 was reduced in the LC-C group compared to the control group (0.53 ± 0.04 *vs.* 0.77 ± 0.02, P < 0.05) (**Figures 2B–D**).

### ERRFI1 Was Up-regulated in A549-IR Cells When Cells Were Treated With LC-C

**Figure 3:**
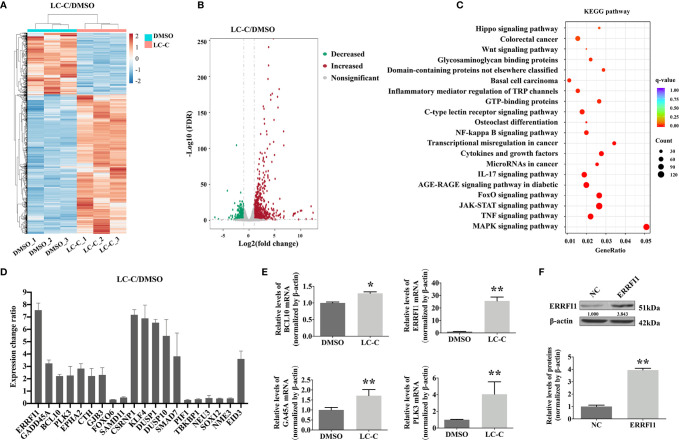
LC-C upregulates ERRFI1 in A549-IR cells. **(A)** Heatmap of RNA-seq analysis showing altered transcriptome profiling in A549-IR cells treated with LC-C. Red color in the heatmap denotes upregulation and blue color denotes down-regulation. **(B)** Volcano plot showing the distribution of -log10 (FDR) *vs*. log2 (Fold Change). Each point represents a gene, red and green points indicate p value < 0.05, while gray points indicate that p value ≥ 0.05. **(C)** KEGG pathways enrichment analysis. **(D)** Graph showing expression changes of the top 20 differentially expressed genes from the RNA-seq analysis; **(E)** RT-qPCR validation of BCL10, ERRFI1, GA45A, and PLK3 upregulation in A549-IR cells after treatment with LC-C. **(F)** Western blotting showing the overexpression of ERRFI1 in A549-IR cells. Results shown are the means ± standard deviation. *Significant difference at P < 0.05 level. **Significant difference at P < 0.01 level.

In order to explore the molecular mechanism of radiosensitization of LC-C on A549-IR cells, we performed RNA-seq analysis using Illumina high-throughput sequencing platform. We detected a total of 1,482 genes differentially expressed in control A549-IR cells (DMSO) *versus* in A549-IR cells with treatment of LC-C (LC-C), including 1,028 up-regulated genes and 454 down-regulated genes for A549-IR cells after LC-C treatment (**Figures 3A, B**). KEGG analysis showed that these differentially expressed genes were enriched in 20 signaling pathways (**Figure 3C**), and most of these pathways were relative to radiosensitivity and cell survival, such as MAPK pathway, NF-*κ*B pathway and JAK/STAT pathway.

Of the top 20 genes that showed the significant differential expression in A549-IR cells after treatment with LC-C (**Figure 3D**), we noticed that four genes, including B cell lymphoma/leukemia-10 (BCL10) (24), ERRFI1 (25–27), growth arrest and DNA damage gene 45A (Gadd45a, GA45A) (28), and Polo-like kinases 3 (PLK3) (29) had been demonstrated to be promising targets for radiation sensitivity. We used RT-PCR to check the changes of the gene expression for these four genes, and the results validated the RNA-seq profiling of these four genes in the A549-IR cells (**Figure 3E**). Of note, both RNA-seq and RT-PCR analyses showed that the ERRFI1 gene was the most significantly up-regulated gene in the A549-IR cells after treatment with 3.5 μM of LC-C.

Furthermore, the clinical significance of ERRFI1 in lung cancer patients was evaluated with Kaplan–Meier Plotter. The median overall survival rate of the high expression cohort was 108.27 months, which was more than that of the low expression cohort (61.30 months) (HR = 0.71, P < 0.001). These results revealed the positive prognostic value of ERRFI1 in lung cancer patients (**Figure S2**).

### LC-C Enhanced Radiosensitivity of A549-IR Cells in an ERRFI1-Dependent Manner

**Figure 4:**
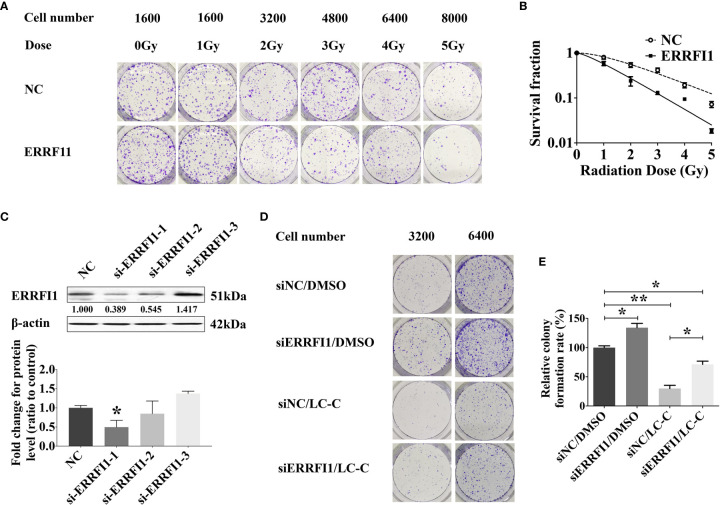
ERRFI1 expression regulates LC-C-enhanced radiosensitivity of A549-IR cells. **(A)** Representative images of colony formation assays in A549-IR cells with/without overexpression of ERRFI1. **(B)** Clonogenic cell survival curves showing the decrease of clonogenic survival in the ERRFI1 overexpressed cells; **(C)** Western blotting showing the ERRFI1 expression after siRNA-ERRFI1 transfection in A549-IR cells, and ERRFI1-1 was used in the subsequent colony formation assays; **(D)** Representative images of colony formation showing the knocking-down of ERRFI1 in A549-IR cells on the radiosensitization effects of LC-C. **(E)** Diagram of clonogenic cell survival rate showing the knocking-down of ERRFI1 in A549-IR cells attenuated radiosensitization effects of LC-C in A549-IR cells. Results shown are the means ± standard deviation. *Significant difference at P < 0.05 level. **Significant difference at P < 0.01 level.

We next tested the potential role of ERRFI1 gene in the radiosensitization effect of LC-C on A549-IR cells. For this, we first engineered A549-IR cells with ERRFI1-overexpression (**Figure 3F**) and then determined the cellular response to irradiation. The colony formation assay showed dramatically decreased clonogenic survival of A549-IR/ERRFI1 cells when compared to the parental control A549-IR cells, with reduced SF2 value from 0.54 ± 0.07 to 0.24 ± 0.06 (p < 0.01), and the sensitizing enhancement ratio of 1.667 (**Figures 4A, B**). We also knocked down ERRFI1 expression with siRNA transfection in A549-IR cells and we found that, although exposure to LC-C significantly enhanced the radiosensitivity of A549-IR cells, ERRFI1 down-regulation attenuated the radiosensitization effect of LC-C on A549-IR cells (**Figures 4C–E**).

**Figure 5:**
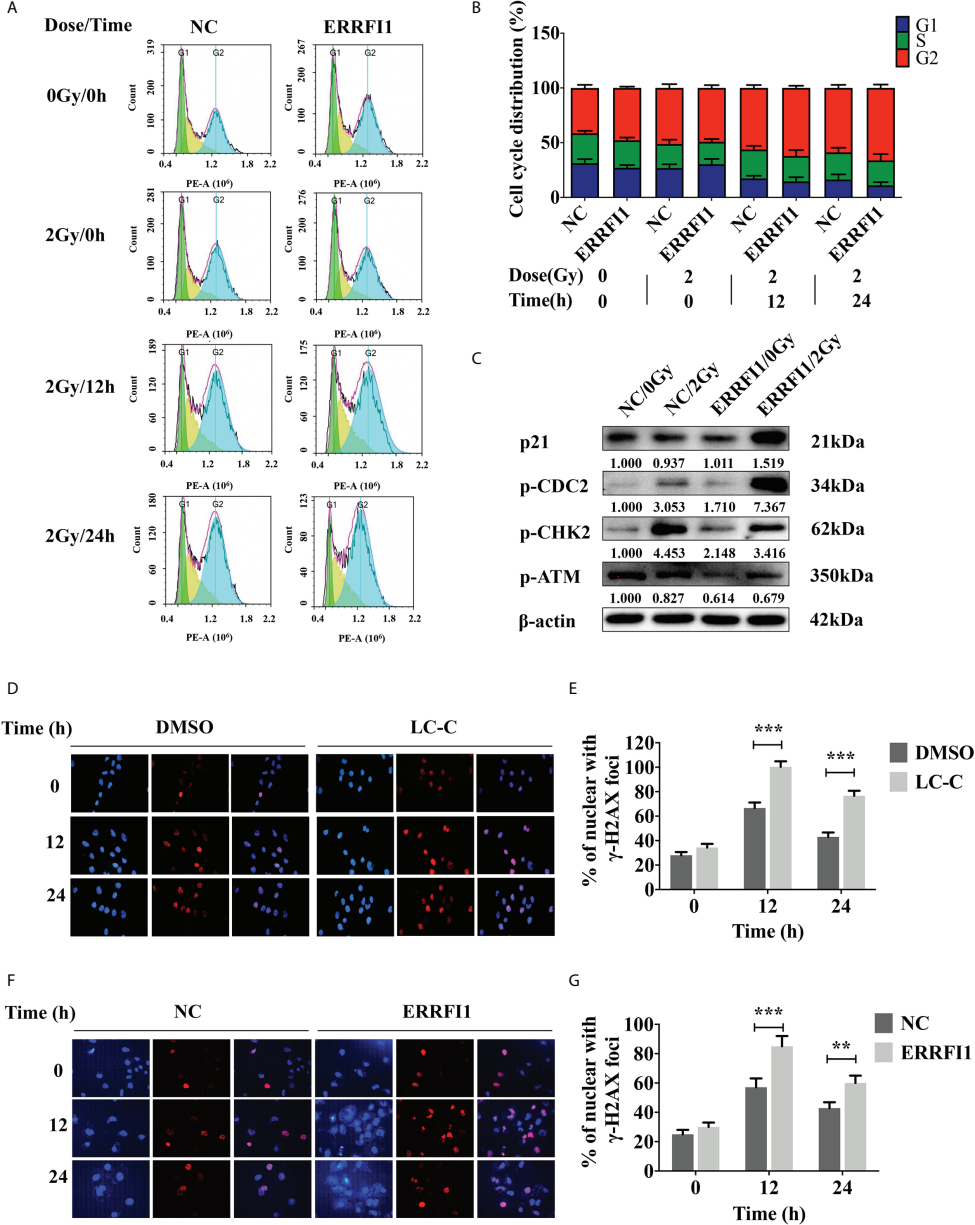
ERRFI1 promotes G2/M cell cycle arrest and LC-C/ERRFI1 blocks IR-induced DNA doublestrand break repair in A549-IR cells after radiation. **(A)** Representative FACS images showing cell cycle distributions of ERRFI1-expressed A549-IR cells after treatment of radiation; **(B)** Histogram of cell cycle distribution of ERRFI1-expressed A549-IR cells after treatment with radiation; **(C)** Western blotting showing the changes of protein expression and phosphorylation in ERRFI1-expressed A549-IR cells after treatment with radiation; **(D)** Representative images of *γ*-H2AX foci in A549-IR cells treated with LC-C and radiation (*γ*-H2AX labeled with red, nuclei with blue). **(E)** Histogram showing the average numbers of *γ*-H2AX foci per cell in A549-IR cells treated with LC-C and radiation. **(F)** Representative images of *γ*-H2AX foci of ERRFI1-expressed A549-IR cells after treatment of radiation (γ-H2AX labeled with red, nuclei with blue). **(G)** Histogram showing the average numbers of *γ*-H2AX foci per cell of ERRFI1-expressed A549-IR cells after treatment with radiation. Results shown are the means ± standard deviations. **Significant difference at P < 0.01 level. ***Significant difference at P < 0.001 level.

We further tested the effects of ERRFI1 on cell cycling in cells in response to irradiation. We used flow cytometry analysis to examine the cell cycle distributions of cells with treatment of 2 Gy irradiation. We found that overexpression of ERRFI1 in A549-IR cells resulted in decreased ratio of the G1/G0 *versus* S phase and an increase of the G2/M phase in response to IR treatment when compared to the control cells (**Figures 5A, B**). Western blot analysis also showed that forced expression of ERRFI1 increased p21 protein expression, enhanced CDC2 phosphorylation (p-CDC2), and reduced CHK2 phosphorylation (p-CHK2) in A549-IR cells when cells were exposed to 2 Gy IR (**Figure 5C**). These results thus indicated that ERRFI1 promoted cell cycle G2/M arrest of A549-IR cells when cells were irradiated.

Immunofluorescence staining for *γ*-H2AX was performed to determine the effect of ERRFI1 on double-strand breaks (DSBs) repair during irradiation. We found that treatment with LC-C increased *γ*-H2AX foci+ cell percentage in A549-IR cells after 2 Gy irradiation for 12 and 24 h (**Figures 5D, E**). Furthermore, our results revealed the significant increases of the *γ*-H2AX foci+ cell percentage in ERRFI1-overexpressed A549-IR cells after 2 Gy irradiation for 12 and 24 h when compared to control cells (**Figures 5F, G**), suggesting a potential of LC-C/ERRFI1 expression blocking DSB repair of irradiated A549-IR cells. To support this, we also detected reduced phosphorylation levels of ATM (p-ATM) and CHK2 (p-CHK2) in ERRFI1-overexpressed A549-IR cells when cells were exposed to 2 Gy IR (**Figure 5C**).

Taken together, these results indicate that the radiosensitization effects of LC-C in A549-IR cells depend on ERRFI1 expression.

### ERRFI1 Expression Reduces the Activation of EGFR and STAT3 Signaling Pathways in Irradiated A549-IR Cells

**Figure 6:**
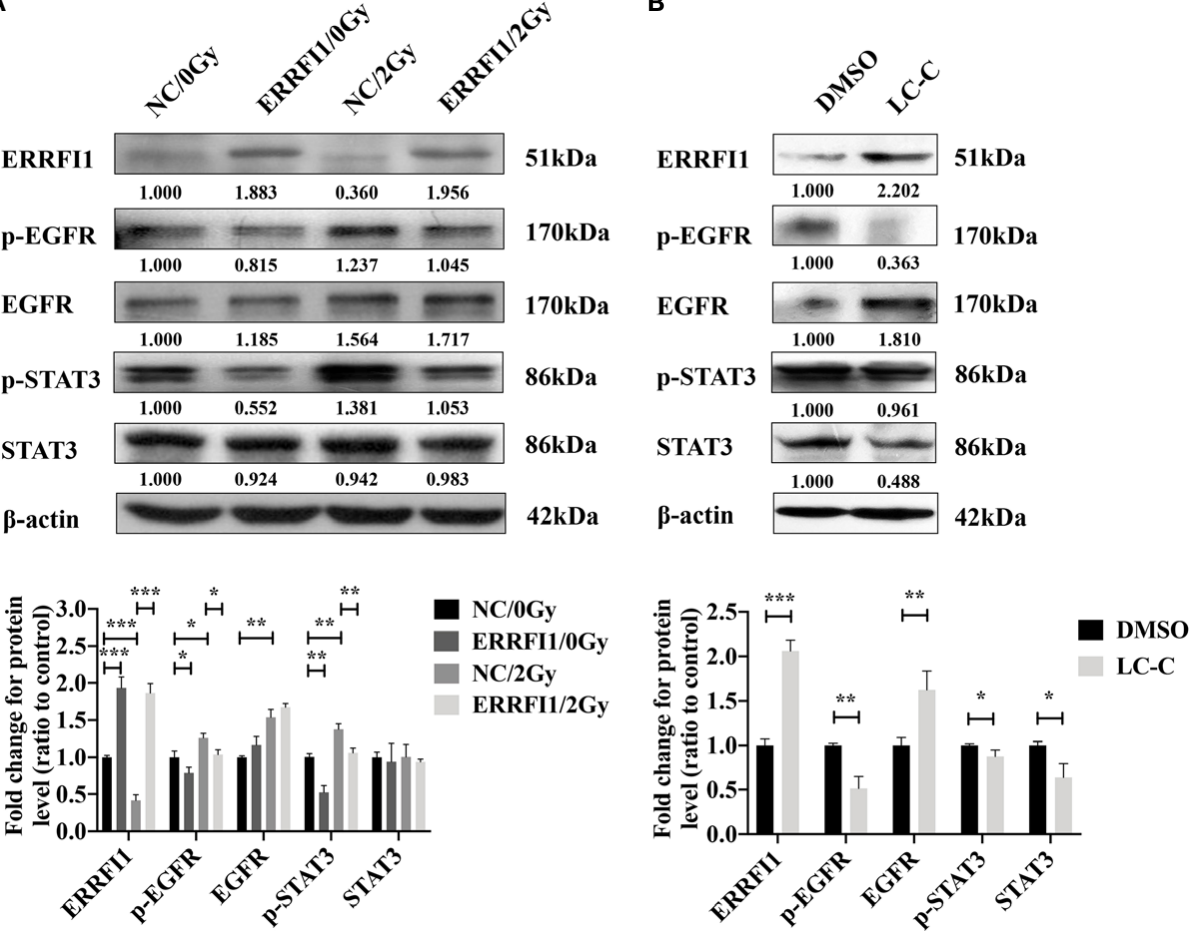
Enhanced radiosensitivity of A549-IR cells by LC-C is associated with decreased activation of EGFR and STAT3 signaling pathways. Western blotting showing the effects of ERRFI1 expression **(A)** or LC-C **(B)** on changes of protein expression and phosphorylation in A549-IR cells. *Significant difference at P < 0.05 level. **Significant difference at P < 0.01 level, ***Significant difference at P < 0.001 level.

To further evaluate the underlying mechanism of how ERRFI1 expression mediates the radiosensitization effects of LC-C, we examined the activation and signaling of EGFR (30) and STAT3. As shown in **Figure 6A**, we detected a moderate increase of p-EGFR and p-STAT3 in A549-IR cells treated with 2 Gy IR. However, we found overexpressing ERRFI1 in A549-IR cells reduced basal level of p-EGFR and p-STAT3 and remarkably inhibited activation of EGFR and STAT3 when cells were exposed to IR. We also noticed treatment with LC-C increased ERRFI1 expression and reduced phosphorylation of EGFR and STAT3 in parental A549-IR cells (**Figure 6B**). These results indicate that the radiosensitization effect of LC-C is associated with ERRFI1-involved EGFR and STAT3 signaling pathways.

### LC-C Enhanced Radiosensitivity in Xenograft Models

**Figure 7:**
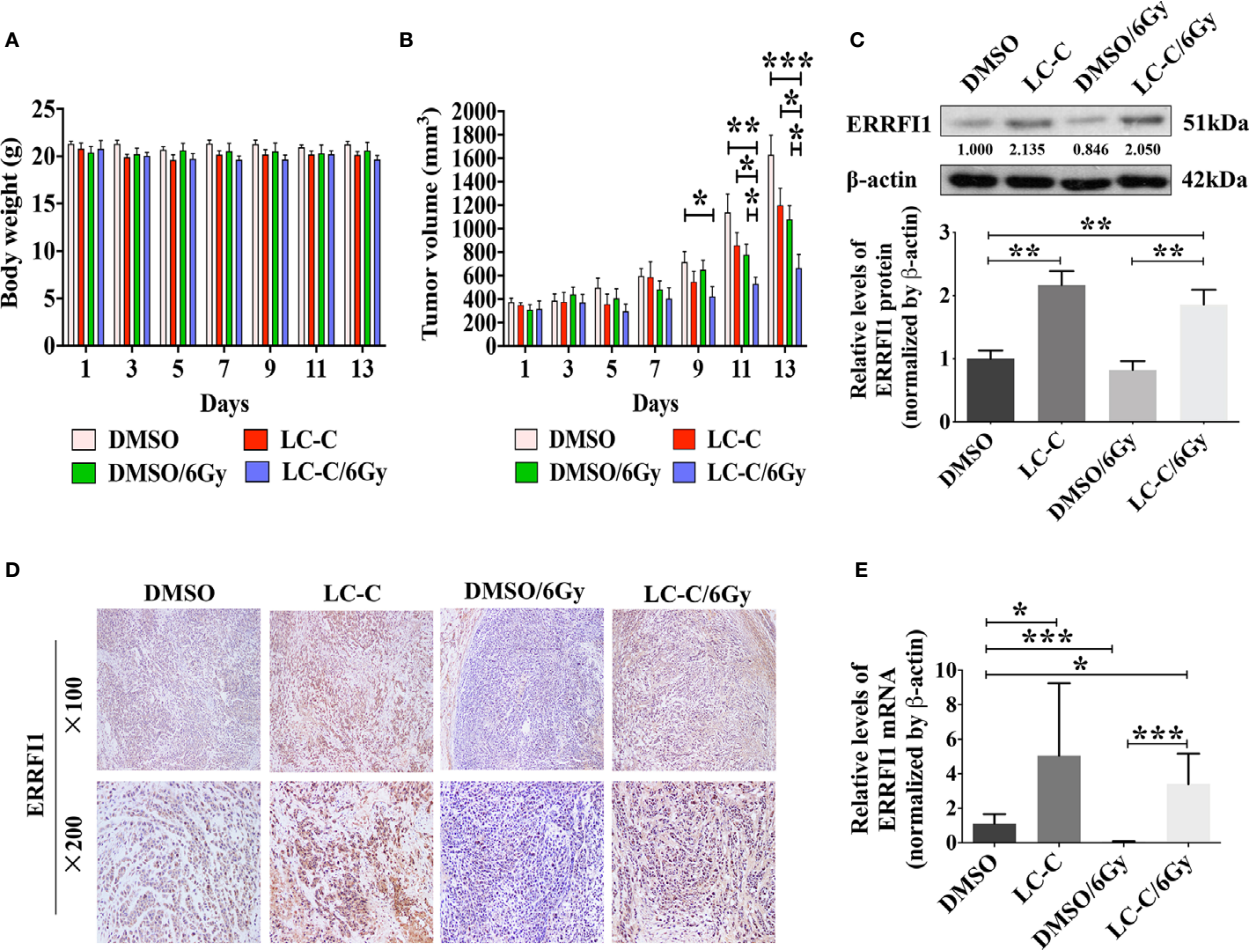
LC-C induces radiosensitivity in xenograft models. A549-IR xenograft nude mice were treated with LC-C, radiation, or both. **(A)** Body weight of mice in four groups; **(B)** Graphs showing the tumor growth of A549-IR xenograft; **(C)** Western blotting showing change of expressions of ERRFI1 in xenograft tumors; **(D)** Immunohistochemical staining of ERRFI1 in xenograft tumors; **(E)** qRT-PCR analysis of ERRFI1 expression in xenograft tumors. Samples were run in triplicate and normalized to *β*-actin mRNA to determine relative expression. One datum that deviated noticeably from the group was eliminated. Results shown are the means ± standard deviation. *Significant difference at P < 0.05 level. **Significant difference at P < 0.01 level. ***Significant difference at P < 0.001 level.

We further used nude mice xenograft model to determine *in vivo* radiosensitization effect of LC-C on ionizing radiation-resistant NSCLC cells. Our results revealed similar body weight of mice in four groups (**Figure 7A**), which suggests that cotreatment with irradiation and LC-C is well tolerated. However, significant growth inhibition on A549-IR xenograft tumors was found in the group with the combination treatment of LC-C/IR when compared to the group with IR or LC-C treatment alone (**Figure 7B**). In addition, the results of immunohistochemical staining, western blotting, and qRT-PCR analysis also confirmed the higher the expression level of ERRFI1 in xenograft tumors treated with the combination of LC-C and IR, compared to radiation alone (**Figures 7C–E**).

## Discussion

Radiation therapy is essential for local tumor control of lung cancer; however, radioresistance is a biological behavior of cancer cells that leads to the failure of radiotherapy and poor prognosis. Some chemotherapeutics are currently used as radiosensitizers, but significant negative side effects still seriously limit their wide application (12). Recently, compounds derived from natural sources have attracted increasing attention due to its anti-cancer activity and acceptable toxicity (13). LC-C, a pentacyclic triterpenoid compound (17), has been proved to have anti-tumor effects in a variety types of cancer; however its radiosensitization potential has not been explored. Herein, our study demonstrated that LC-C could sensitize ionizing radiation-resistant lung cancer cells to IR treatment *in vitro* and *in vivo*, and the radiosensitization effects of LC-C correlated to the level of ERRFI1 expression.

The ERRFI1 gene locus is on chromosome 1p36, a segment of chromosome where many cancer-related genes are located (25). ERRFI1 is a tumor suppressor gene (24), and low ERRFI1 expression has been observed in various types of cancer (25, 31). In breast cancer, down-regulation of ERRFI1 expression is correlated with poor survival (32). Based on Kaplan**–**Meier Plotter, we found that the expression level of ERRFI1 is associated with a positive prognosis in lung cancer patients. Besides, ERRFI1 is a transcriptional target of epidermal growth factor (EGF) (33), and as a feedback, ERRFI1 expression inhibits tyrosine phosphorylation of EGFR (34) and the downstream activation of ERK, JNK, and Akt signaling (35). ERRFI1 binds to EGFR family member proteins *via* its ErbB2-binding domain and inhibits the tyrosine kinase activity. Consequently, loss of ERRFI1 expression in human lung cancer cells showed no response to the activated EGF pathway (25). Interestingly, activation of EGFR signaling pathway plays important roles in radiosensitivity of cancer cells as irradiation induces unusual activation of EGFR signaling and thus causes radioresistance through EGFR activation-controlled cell proliferation, DNA repair, hypoxia, and metastasis (36). Up to now, there have been no reports on the radiosensitization effect of ERRFI1 gene. Our results showed decrease of ERRFI1 protein expression and increased of p-EGFR protein expression in A549-IR cells when cells were treated with radiation, suggesting the significant decreased expression of ERRFI1 was associated with the abnormal activation of EGFR signaling pathway and the invalidation of the negative feedback regulation mechanism.

STAT3 is also a critical transcriptional regulatory target of EGFR, and its activation regulates DNA damage repair, autophagy, apoptosis, proliferation, angiogenesis, migration, invasion, and immune suppression in lung cancer cells (37–39). Our results showed that the expression level of p-STAT3 protein was increased in A549-IR cells treated with radiation alone and decreased in A549-IR cells overexpressed with ERRFI1, which revealed that EGFR/STAT3 signaling pathway may be involved in the radiosensitization of ERRFI1.

DSBs are the main form of damage resulting in cell death after irradiation, and DNA damage responses, including repair, and checkpoint activation after DSBs are important molecular mechanisms of radiotherapy sensitivity. Phosphorylation of *γ*-H2AX was extensively studied as a sensitive indicator of DSBs after radiation (40). Consequently, observation and counting of *γ*-H2AX foci under fluorescence microscopy may indicate a complexity of DNA damage. In our study, higher level of *γ*-H2AX and reduced phosphorylation levels of DNA damage repair protein, including p-ATM and p-CHK2, in ERRFI1-overexpressed group than that in the control group suggest that prolonged process of DNA damage repair could contribute to ERRFI1-mediated radiosensitization in lung cancer cells. In addition, we found that ERRFI1-overexpression significantly enhanced radiation-induced G2/M arrest and also increased p21, p-CDC2 expression, reduced p-CHK2 expression in lung cancer cells. Since G2/M phase cells have the highest radiosensitivity, the high radiosensitivity associated with ERRFI1 may be due to the G2/M arrest of A549-IR cells, leaving most cells in the G2/M phase, thus causing a large number of cells to be killed. These results may help in understanding the detailed molecular mechanisms of how ERRFI1 contributes to radiation sensitivity of NSCLC cells.

In this study, we investigated for the first time the effect of LC-C pt?>on tumor cell radiosensitivity of lung cancer cells. Our results showed that ERRFI1 was up-regulated in lung cancer cells after treatment with LC-C *in vitro* and *in vivo*, and the expression level of p-EGFR and p-STAT3 was decreased in A549-IR cells after treatment with LC-C, which revealed that ERRFI1 and EGFR/STAT3 may be critical molecules in the radiosensitization effect of LC-C. LC-C may promote radiation induced anti-tumor effect by increasing the expressions of ERRFI1 and associated with EGFR and STAT3 signaling pathways.

Further investigations are warranted to determine the regulation mechanism of LC-C on ERRFI1, which might contribute to develop more drugs for enhancing radiosensitivity of lung cancer. Tumor suppressor genes can be inactivated by somatic mutation and/or promoter methylation (41). Previous studies showed that genetic mutations in the ERRFI1 coding region do not seem to be frequent in the cancers analyzed (25). Conversely, as many CpG islands presented in the ERRFI1 promoter region (42), DNA methylation may be a promising direction for future research on the radiosensitivity mechanism of LC-C.

## Conclusions

The current study demonstrated the LC-C sensitized ionizing radiation-resistant lung cancer cells to radiation both *in vitro* and *in vivo*, and the radiosensitization effect was attributed to the up-regulation of ERRFI1 and the down-regulation of EGFR/STAT3 pathway. Our results provide a rationale for future clinical investigation of the therapeutic efficacy of LC-C in combination with radiotherapy. However, the detailed mechanism of LC-C upregulating the expression of ERRFI1 remains unclear, and further studies are needed.

## Data Availability Statement

The sequencing data supporting the conclusions of this article are available on Figshare (https://figshare.com/articles/dataset/RNA_sequencing_results_xls/13489116).

## Ethics Statement

The animal study was reviewed and approved by Animal Care and Use Committee, Zhejiang Chinese Medical University.

## Author Contributions

SM and BX conceived and supervised the project. KW and XC performed the experiments. SM, JF, and SZ collected data. BX, JZ, and QW analyzed the data. KW, YX, and MY wrote the manuscript. YX, KW, and BX critically revised the paper. All authors contributed to the article and approved the submitted version.

## Funding

This work was supported by the Natural Science Foundation of Zhejiang Province (Grant No. LQ20H160019, LQ17H160003), National Natural Science Foundation of China (Grant No. 81803042) Zhejiang Traditional Chinese Medicine Research Project (Grant No. 2020ZB195), and Zhejiang Provincial Traditional Chinese Medicine Science and Technology Project (Grant No.2018ZY009).

## Conflict of Interest

The authors declare that the research was conducted in the absence of any commercial or financial relationships that could be construed as a potential conflict of interest.

## Supplementary Material

Supplementary Figure 1 Chemical structure of LC-C.

Supplementary Figure 2 The overall survival rates of lung cancer patients with high ERRFI1 level and low ERRFI1 level were evaluated with Kaplan–Meier Plotter. The high expression level of ERRFI1 was associated with high overall survival rates.
