# Supplementary figures and images for: Capilliposide C from Lysimachia capillipes Restores Radiosensitivity in Ionizing Radiation-Resistant Lung Cancer Cells Through Regulation of ERRFI1/EGFR/STAT3 Signaling Pathway
Source: Front Oncol. 2021 Apr 1;11:644117. doi: 10.3389/fonc.2021.644117 (PMC8047471; doi:10.3389/fonc.2021.644117)

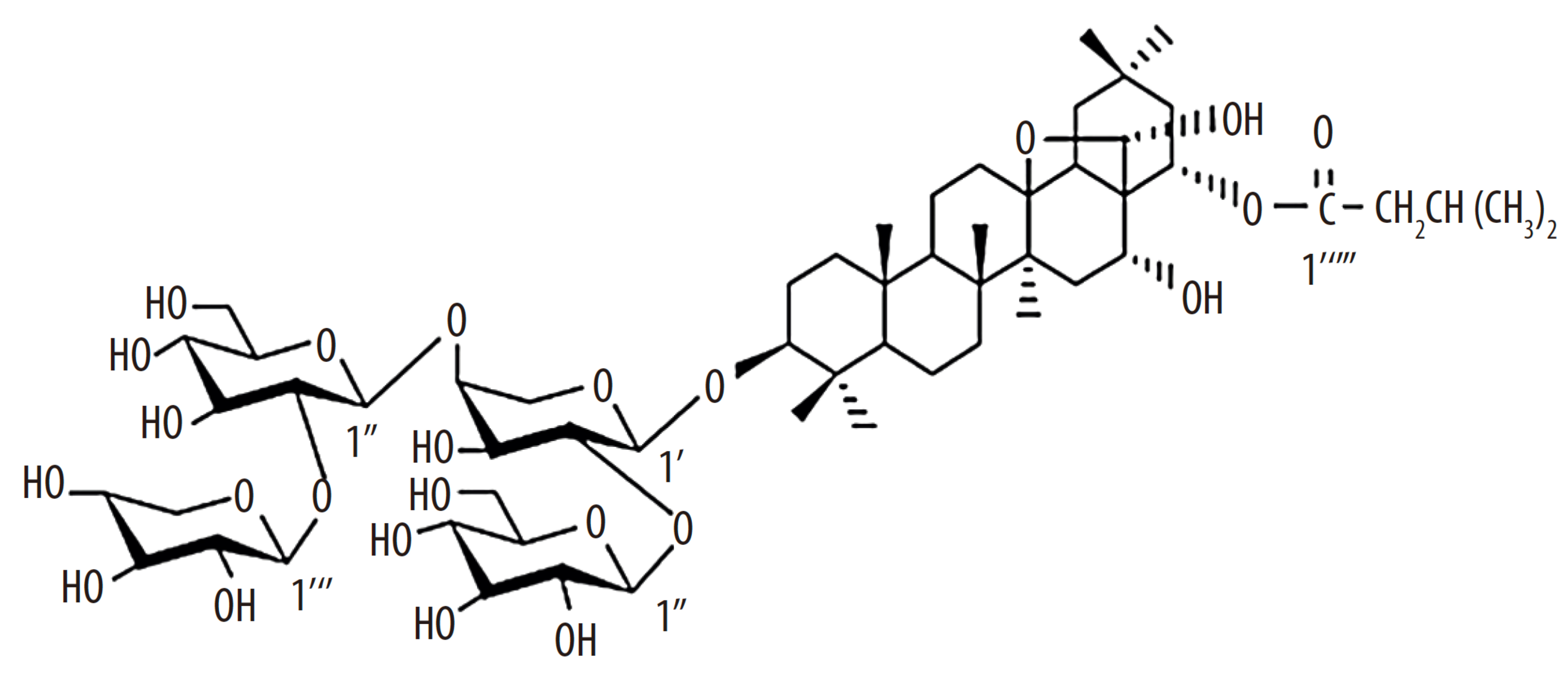

Supplement: Supplementary Figure 1 — Chemical structure of LC-C. [file Image_1.jpeg]

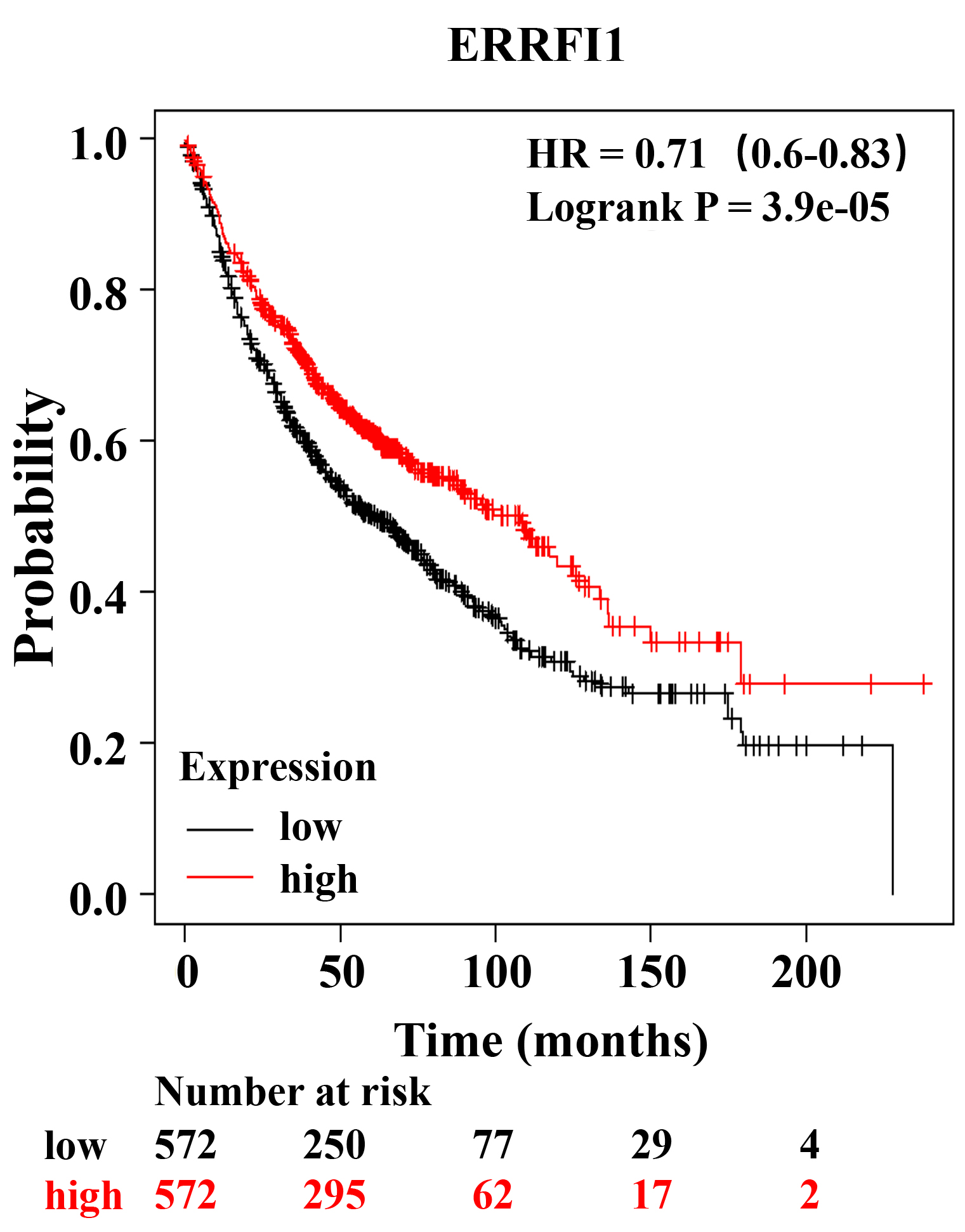

Supplement: Supplementary Figure 2 — The overall survival rates of lung cancer patients with high ERRFI1 level and low ERRFI1 level were evaluated with Kaplan–Meier Plotter. The high expression level of ERRFI1 was associated with high overall survival rates. [file Image_2.jpeg]
